# The alternatively spliced fibronectin CS1 isoform regulates IL-17A levels and mechanical allodynia after peripheral nerve injury

**DOI:** 10.1186/s12974-015-0377-6

**Published:** 2015-09-04

**Authors:** Huaqing Liu, Jennifer Dolkas, Khan Hoang, Mila Angert, Andrei V. Chernov, Albert G. Remacle, Sergey A. Shiryaev, Alex Y. Strongin, Tasuku Nishihara, Veronica I. Shubayev

**Affiliations:** Department of Anesthesiology, University of California, 9500 Gilman Dr., Mail Code 0629, La Jolla, San Diego, CA 92093-0629 USA; VA San Diego Healthcare System, La Jolla, CA USA; Sanford-Burnham Medical Research Institute, La Jolla, CA USA

**Keywords:** Fibronectin, CS1, IL-17, Neuropathic, Pain, Allodynia, T cell, Th17, A-afferent, Myelin, Schwann cell

## Abstract

**Background:**

Mechanical pain hypersensitivity associated with physical trauma to peripheral nerve depends on T-helper (Th) cells expressing the algesic cytokine, interleukin (IL)-17A. Fibronectin (FN) isoform alternatively spliced within the IIICS region encoding the 25-residue-long connecting segment 1 (CS1) regulates T cell recruitment to the sites of inflammation. Herein, we analyzed the role of CS1-containing FN (FN-CS1) in IL-17A expression and pain after peripheral nerve damage.

**Methods:**

Mass spectrometry, immunoblotting, and FN-CS1-specific immunofluorescence analyses were employed to examine FN expression after chronic constriction injury (CCI) in rat sciatic nerves. The acute intra-sciatic nerve injection of the synthetic CS1 peptide (a competitive inhibitor of the FN-CS1/α4 integrin binding) was used to elucidate the functional significance of FN-CS1 in mechanical and thermal pain hypersensitivity and IL-17A expression (by quantitative *Taqman* RT-PCR) after CCI. The CS1 peptide effects were analyzed in cultured primary Schwann cells, the major source of FN-CS1 in CCI nerves.

**Results:**

Following CCI, FN expression in sciatic nerve increased with the dominant FN-CS1 deposition in endothelial cells, Schwann cells, and macrophages. Acute CS1 therapy attenuated mechanical allodynia (pain from innocuous stimulation) but not thermal hyperalgesia and reduced the levels of IL-17A expression in the injured nerve. CS1 peptide inhibited the LPS- or starvation-stimulated activation of the stress ERK/MAPK pathway in cultured Schwann cells.

**Conclusions:**

After physical trauma to the peripheral nerve, FN-CS1 contributes to mechanical pain hypersensitivity by increasing the number of IL-17A-expressing (presumably, Th17) cells. CS1 peptide therapy can be developed for pharmacological control of neuropathic pain.

**Electronic supplementary material:**

The online version of this article (doi:10.1186/s12974-015-0377-6) contains supplementary material, which is available to authorized users.

## Introduction

Neuropathic pain, a severe pain state arising from a lesion or disease of the nervous system, is refractory to analgesics [1]. A rodent model of chronic constriction injury (CCI) to the sciatic nerve allows to study the two major neuropathic pain phenotypes of exaggerated pain from noxious thermal stimulus (thermal hyperalgesia) and pain evoked by innocuous mechanical stimulus (mechanical allodynia). Current understanding of the neuropathic pain mechanisms recognizes the hyperalgesic activity of inflammatory cytokines and chemokines, produced initially by the resident cells of the nerve, including Schwann cells, macrophages, and endothelial cells. These inflammatory mediators contribute to both the depolarization of nociceptors and the chemotactic gradients that guide hematogenous monocytes and leukocytes into the damaged nerve [2–8]. Sustained activity of the innate immune system subsequently activates antigen-specific adaptive immunity, which engages T helper (Th)1 and Th17 cells expressing pro-inflammatory/algesic interleukin (IL)-1β and IL-17A, respectively, to sustain neuropathic pain states that follow nerve trauma [4, 9–17]. Conversely, Th2 and Treg(ulatory) cells express anti-inflammatory/analgesic cytokines and traffic to the injury site in order to reduce pain [4, 9–17].

Differential regulation of mechanical but not thermal (heat) hypersensitivity involves adaptive immunity modulators, such as IL-17A [13], IL-4 [18], toll-like receptor 4 [19], myelin basic protein [17], and fibronectin (FN) [20], and engages low-threshold mechanosensory myelinated A-afferents [21, 22]. In contrast, inflammatory modulators universally expressed by various immune and glial cells, including TNF-α, IL-1β, and IL-6, indiscriminately modulate thermal and mechanical pain hypersensitivity [2–8] by stimulating heat-nociceptive C-afferents and mechanosensitive A-afferents, respectively [23, 24]. According to our recent findings, the proteolysis of the insulating myelin sheath of A-afferents results in the release of the cryptic myelin auto-antigens, which stimulate the development of mechanical allodynia in part via Th17 cell homing to myelinated fibers [17]. The molecular events underlying Th17 cell homing in the injured nerve and their emerging functions in nociceptive circuits remain obscure.

T cells gain access into the sites of inflammation via the integrin α4β1 binding to the connecting segment 1 (CS1) isoform of FN. This isoform is alternatively spliced within the IIICS [or variable (V)] region encoding the 25-residue-long CS1 peptide sequence (Asp-Glu-Leu-Pro-Gln-Leu-Val-Thr-Leu-Pro-His-Pro-Asn-Leu-His-Gly-Pro-Glu-Ile-Leu-Asp-Val-Pro-Ser-Thr) [25–28]. FN is an adhesion glycoprotein and a component of the extracellular matrix that, among its multiple functions, provides architectural scaffolding to regenerating axons [29, 30]. Alternative splicing of the EIIIA, EIIIB, and IIICS exons of the FN gene in nerves leads to the presence of the multiple FN isoforms [31, 32]. Regardless of the abundance of the CS1-containing FN (FN-CS1) isoform in the peripheral nerve and cultured Schwann cells [31, 32], the pathophysiological role of FN-CS1 in T cell recruitment post-nerve injury is not well understood. Typically, interference with the FN-CS1 binding to the α4β1 integrin using the competitively blocking CS1 peptide abrogates T cell homing to inflamed tissues [26, 28, 33, 34].

Herein, we have established that FN-CS1 controls the content of IL-17A expressing (presumably Th17) cells and mechanical allodynia in rats undergoing CCI. Conversely, acute therapeutic intervention that employs the synthetic CS1 peptide attenuates mechanical allodynia, presenting a likely valuable strategy to control the algesic IL-17A action and mechanical pain hypersensitivity phenotype associated with nerve trauma.

## Materials and methods

### Animal model

All animal procedures were performed according to the PHS Policy on Humane Care and Use of Laboratory Animals and the protocols approved by the Institutional Animal Care and Use Committee at the VA San Diego Healthcare System and complied with ethical guidelines of the International Association for the Study of Pain. Female Sprague-Dawley rats (*n* = 53, 200–225 g, Harlan Labs) were housed in a temperature-controlled room (at 22 °C) on a 12-h light/dark cycle with free access to food and water. Animals were anesthetized with 4 % isoflurane (Aerrane; Baxter) in 55 % oxygen. The common sciatic nerve was exposed unilaterally and received three loosely constrictive chromic gut ligatures to produce CCI [35]. Naïve animals were used for control. All animals were sacrificed using Beuthanasia i.p. (Schering-Plough Animal Health), and sciatic nerve segments were collected for analyses.

### CS1 peptide therapy

The N-end acetylated and C-end amidated wild-type CS1 (DELPQLVTLPHPNLHGPEILDVPST) and scrambled CS1 (sCS1, EPDELQTGHVLSPLNHTPVLIPLDP) peptides were synthesized by GenScript. Immediately after CCI, CS1 and sCS1 (50 μg/ml in 5 μl PBS) or PBS alone (5 μl) were injected into the nerve fascicle (injury site) using a Hamilton syringe with a 33-gauge needle.

### Pain-like behaviors

Animals were habituated to the testing environment prior to baseline tests. For assessment of *mechanical hypersensitivity*, rats were placed in individual plexiglass compartments with wire mesh bottom, and after acclimatization, von Frey filaments (0.41–15.2 g, Stoelting) were applied perpendicularly to the mid hind paw and held for 4–6 s. A positive response was noted if the paw was sharply withdrawn. The 50 % probability of withdrawal threshold was determined by Dixon’s up-down method [36]. To assess *thermal hypersensitivity*, a modified Hargreaves-type device was employed [37]. Rats were placed individually in plexiglass cubicles with a glass surface, and after habituation, a radiant heat stimulus was applied to each paw, and the latency defined as the time (seconds) required for the paw to show a brisk withdrawal. Tests were performed for 3 days before CCI and then at days 1, 2, 3, 5, and 7 after CCI and therapy by an examiner blinded to the experimental groups.

### Neuropathology

Sciatic nerve segments were excised and post-fixed for 48 h at 4 °C using 2.5 % glutaraldehyde in 0.1 M phosphate buffer, pH 7.4. Specimens were washed using phosphate buffer, post-fixed with 1 % osmic acid (Ted Pella), dehydrated in graded (30–100 %) ethyl alcohol and propylene oxide, and embedded in Araldite resin (Ted Pella). One-micron-thick sections were cut using a diamond knife in an automated RM2065 microtome (Leica Microsystems) and stained using methylene blue/azure II solution [38]. Sections from three animals per group and three randomly selected areas per section were analyzed.

### Immunofluorescence

Sciatic nerve segments were excised, post-fixed in 4 % p-formaldehyde, cryoprotected in a 15–30 % sucrose gradient, embedded into the optimal cutting temperature (OCT) compound (Sakura Finetek) in liquid nitrogen, and cut into 10-μm-thick transverse sections. Non-specific binding was blocked using 10 % normal goat serum. The slides were incubated for 16–18 h at 4 °C with mouse anti-human CS1 antibody (EMD Millipore, cat. #MAB1939), followed by incubation for 16–18 h at 4 °C with Alexa Fluor 594-conjugated goat anti-mouse IgM (Life Technologies, cat. #A21042). For dual immunofluorescence, following the washes in PBS-1 % Tween, the slides were incubated for 1–2 h at ambient temperature with rabbit polyclonal CD68 antibody (Santa Cruz, cat. #SC9139), followed by incubation with goat anti-rabbit Alexa 488-conjugated secondary antibody (green, 1–2 h, ambient temperature).

Teased nerve fibers were prepared from the separated nerve bundles using fine smooth microforceps and incubated in PBS-5 % fish skin gelatin-0.1 % Triton X-100 for 1 h. After the staining described above, individual fibers were teased out on a glass slide using a 0.20- to 0.22-mm acupuncture needle (Vinco, Oxford Medical Supplies) for the follow-up observation. Slides were mounted using the Slowfade Gold antifade reagent containing 4′,6-diamidino-2-phenylindole (DAPI; Molecular Probes). Signal specificity was confirmed by omitting the primary antibody. The images were acquired using a Leica DMRB microscope and Openlab 4.04 software (Improvision).

### Immunoblotting

Sciatic nerve and Schwann cell extracts were prepared using 50 mM Tris-HCl, pH 7.4, containing 1 % Triton X-100, 150 mM NaCl, 10 % glycerol, 0.1 % SDS, 5 mM EDTA, 1 mM phenylmethylsulfonyl fluoride, aprotinin, and leupeptin (1 μg/ml each). The protein concentration of the extracts was determined using bicinchoninic acid assay. Following reduction, aliquots of tissue and cell extracts (40 μg and 10–15 μg each, respectively) were separated by SDS-PAGE in a 5–12 % gradient gel. Proteins were transferred onto a nitrocellulose support using an iBlot dry blotting system (Invitrogen) at 20 V for 7 min. The membranes were blocked using TBS-0.1 % Tween-20–5 % non-fat milk (Bio-Rad) and incubated overnight at 4 °C with mouse FN antibody (Santa Cruz, cat. #SC8422), rabbit phospho-ERK1/2 antibody (Thr202/Tyr204, Cell Signaling, cat. #9101), or rabbit ERK1/2 antibody (Cell Signaling, cat. #9102) followed by incubation for 1 h at ambient temperature with horseradish peroxidase-conjugated goat anti-mouse or anti-rabbit secondary antibodies (Cell Signaling). The blots were developed using an enhanced chemiluminescence system (GE Healthcare). For loading control, the membranes were re-probed using mouse β-actin antibody (Sigma, cat. #A53166). The band density was measured in *n* = 3/group relative to that of β-actin using Image J Software.

### qRT-PCR

Sciatic nerves were isolated and stored at −20 °C in RNA-later (Ambion). Primers and *Taqman* probes for IL-17А (GenBank #NM_001106897) were obtained from Applied Biosystems (Assay ID Rn01757168_m1) and for glyceraldehyde 3-phosphate dehydrogenase (GAPDH, GenBank #X02231) from Biosearch Technologies [17]. Total cell RNA was extracted using TRIzol (Invitrogen) and purified using an RNeasy mini column (Qiagen). The RNA purity was estimated by measuring the OD260/280 ratio. The samples were treated with RNase-free DNAse I (Qiagen). cDNA was synthesized using a SuperScript first-strand RT-PCR kit (Invitrogen). Gene expression was measured using a Mx4000™ Multiplex Quantitative PCR System (Agilent Technologies) with 50 ng cDNA and 2× *Taqman* Universal PCR Master Mix (Ambion). A one-step amplification program (95 °C, 10 min; 95 °C, 30 s; 60 °C, 1 min) was normally used for 50 cycles. For naïve nerve samples that do not exhibit any IL-17A signal, a threshold cycle (Ct) value of 51 was assigned to allow calculations. Duplicate samples missing cDNA (a “no template” control) showed no contaminating DNA. Relative mRNA levels were quantified using the 2(-Delta Delta C(T)) method [39]. Normalization to GAPDH and fold-change calculations were performed using MxPro software (Agilent Technologies).

### Two-dimensional liquid chromatography/tandem mass spectrometry/mass spectrometry (2D-LC/MS/MS)

Sciatic nerves were isolated, snap-frozen in liquid nitrogen, and stored at −80 °C. The samples were homogenized, sonicated, and extracted 60 min at ambient temperature in 100 mM Tris-HCl, pH 8.0, containing 8 M urea and the protease and phosphatase inhibitor cocktail. The insoluble material was removed by centrifugation (16,000×*g*; 15 min). The supernatant samples (at least 0.5 mg total protein each) were then processed by the Proteomics Core facility of the Sanford-Burnham Medical Research Institute. The samples were reduced (10 mM tris(2-carboxyethyl) phosphine, 37 °C, 30 min), alkylated (20 mM iodoacetamide, 37 °C, 40 min in the dark), and digested using modified trypsin, mass spectrometry grade (Promega; 1:100 *w*/*w* ratio; 37 °C, 16–18 h). The samples were desalted using a SepPack cartridge, dried using a SpeedVac, and re-suspended in 0.1 ml 5 % formic acid. The resulting peptides were separated into 24 fractions using an offline Michrom MDLC pump (Michrom) with a Michrom Strong Cation Exchange column. The 1/10 aliquot of each peptide fraction was analyzed using an LTQ-Orbitrap XL mass-spectrometer (Thermo Scientific) and a 15-cm Michrom Magic C18 column coupled with a low-flow Michrom ADVANCED device. The data were analyzed by Sorcerer Enterprise v.3.5 software (Sage-N Research) using the ipi.Rat.v3.56 protein database. To identify carboxyamidomethylated cysteines, 57 Da were added to cysteines, and 16 Da were added to methionines to identify oxidated methionines. The search results were sorted, filtered, and statistically analyzed using a trans-proteomic pipeline (TPP) (Institute for Systems Biology) with a 90 % minimum probability score and an error rate ≤2 %. An additional, confirmatory search was performed using a Prolucid search algorithm with a DTASelect function via an Integrated Proteomics Pipeline (IP2) server.

### Stimulation of cultured Schwann cells

Primary Schwann cell cultures were obtained using the Brockes method [40]. Sciatic nerves of postnatal day 1–3 Sprague-Dawley rats (Harlan Labs) were isolated and purified using AraC, an anti-fibronectin Thy1.1 antibody and the rabbit complement [38, 41]. The purity of the obtained cultures was confirmed by the Schwann-cell-specific S100B immunopositivity (>99 %). Schwann cells were re-suspended and plated in poly-d-lysine-coated dishes in Dulbecco’s modified Eagles medium (DMEM), containing 10 % fetal bovine serum (FBS), 100 units/ml penicillin and 100 μg/ml streptomycin, 21 μg/ml bovine pituitary extract, and 4 μM forskolin (referred to as “complete medium”) at 37 °C under humidified 5.0 % CO_2_. Cells of passage 3–7 were grown in a 6-well-dish in a complete medium until reaching 70 % confluence. Cells were then starved in DMEM-1 % FBS for 24 h and treated with lipopolysaccharide (LPS; 100 ng/ml) from *Escherichia coli* (Sigma, cat. # L2880) for 15 min at 37 °C. CS1 (2.5 μg/ml) was added into the medium, and cells were incubated at 37 °C. In 15–60 min, cells were washed twice at 37 °C using pre-warmed TBS. Cell extracts were prepared using TBS supplemented with 1 % Triton X-100, 0.5 % sodium deoxycholate, 0.1 % SDS, protease inhibitor cocktail, and 1 mM sodium orthovanadate. The prepared extracts were subjected to immunoblotting. Data was obtained from three independent experiments.

### Data analyses

Statistical analyses were performed using KaleidaGraph 4.03 (Synergy Software) and InStat 3 (GraphPad Software) using two-tailed, unpaired Student’s *t*-test for comparing two groups or analyses of variance (ANOVA) for repeated measures for comparing three or more groups, followed by Tukey-Kramer post hoc test. *p* ≤ 0.05 values were considered significant.

## Results

### FN-CS1 expression in CCI nerve

**Fig. 1:**
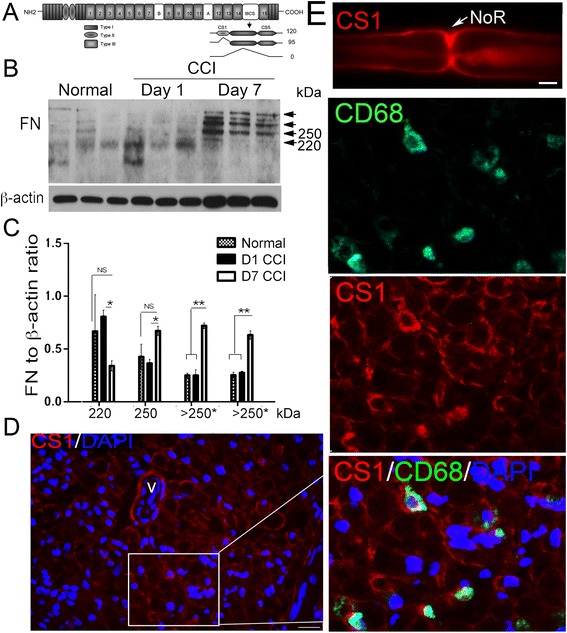
FN in CCI nerve. **a** Fibronectin (FN) exists as a heterodimer linked by disulfide bonds. One of the monomers includes the 25-amino-acid-long CS1 sequence. Typical CS1 inclusion and exclusion splice variants are illustrated. **b** FN immunoblot of rat sciatic nerve, normal or at days 1 and 7 after CCI. β-actin, a loading control (40 μg total protein). **c** The graph represents the mean levels of the individual FN isoform recorded in (**b**). ±SEM relative to β-actin (*n* = 3–4/group; *p < 0.05, **p < 0.01, *n.s.* not significant). **d** FN-CS1 immunostaining (*red*) in sciatic nerves at day 7 post-CCI. The vessel (*V*) endothelial cells, Schwann cells, and macrophages (confirmed using the CD68 immunostaining, *green*) are stained. DAPI, *blue*. Representative of *n* = 3/group; scale bars, 40 μm. **e** FN-CS1 immunostaining (*red*) in a teased nerve fiber at day 7 post-CCI; representative of ~20 individual fibers from *n* = 3. Scale bar, 10 μm. *NoR* node of Ranvier

As schematically illustrated in Fig. 1a, FN is a dimer of the 220–250-kDa nearly identical monomers linked via disulfide bonds and encoded by a single FN transcript [30]. The FN transcript undergoes alternative splicing within the three independent exons, EIIA, EIIIB, and IIICS (also known as variable, V) [42]. The V site produces no inserts (called *V*0) or two inserts of 95 or 120 amino acids (called V95 and V120, respectively, Fig. 1a) differing by a 25-amino-acid CS1 segment [42]. In agreement, the dominant 220-kDa band of the FN monomer and additional species with the molecular weight over 250 kDa were observed in the normal nerve (Fig. 1b, c). Concomitantly, the levels of the 220-kDa FN slightly decreased while alternatively spliced FN species with the molecular weight over 250 kDa were elevated at day 1 and especially day 7 post-CCI (*p* < 0.05), during T cell recruitment [9–12].

To support these observations, the protein extracts obtained from the CCI and sham-operated sciatic nerves were subjected to 2D-LC/MS mass spectrometry analysis. For these purposes, extracted proteins were fully denatured, reduced, alkylated, and digested by trypsin. The resulting trypsin fragments of the nerve proteome were separated by liquid chromatography (LC), and each fraction was next analyzed using a mass spectrometer to determine the identity of the peptides. Our in-detail analysis unambiguously identified a number of the FN peptides in the nerve samples (Table 1). The length of the identified peptides if combined represented 35 % of the FN protein sequence (35 % coverage). Because of both the limited difference in the expression levels of the FN isoforms in the injured versus intact nerve [31, 32, 43] and the incomplete, albeit sufficient, coverage of the FN sequence, the detected peptides are grouped together. The presence of the T*DELPQLVTLPHPNLHGPEI****LDV****PS*TVQK 2081–2109 peptide that included the CS1 sequence (italicized; the LDV sequence that is essential for the binding to α4β1 integrin is in bold) implied that the 2477-residue-long rat FN-CS1 splice variant was present in CCI nerve.

**Table 1:** FN peptide sequences in the rat sciatic nerve

	Peptide sequence		Peptide sequence
1	^69^TYLGNALVCTCYGGSR^84^	27	^1446^TGLDSPTGFDSSDVTANSFTVHWVAPR^1472^
2	^85^GFNCESKPEPEETCFDK^101^	28	^1473^APITGYIIR^1481^
3	^85^GFNCESKPEPEETCFDKYTGNTYK^108^	29	^1525^EESPPLIGQQSTVSDVPR^1542^
4	^118^DSMIWDCTCIGAGR^131^	30	^1570^ITYGETGGNSPVQEFTVPGSK^1590^
5	^118^DSMIWDCTCIGAGRGRISCTIANR^141^	31	^1591^STATINNIKPGADYTITLYAVTGR^1614^
6	^254^GNLLQCVCTGNGR^266^	32	^1615^GDSPASSKPVSINYQTEIDKPSQMQVTDVQDNSISVR^1651^
7	^274^HVLQSASAGSGSFTDVR^290^	33	^1652^WLPSTSPVTGYR^1663^
8	^291^TAIYQPQTHPQPAPYGHCVTDSGVVYSVGMQWLK^324^	34	^1708^NGESQPLVQTAVTNIDRPK^1726^
9	^370^TFYSCTTEGR^379^	35	^1727^GLAFTDVDVDSIK^1739^
10	^380^QDGHLWCSTTSNYEQDQK^397^	36	^1820^FTQVSPTTLTAQWTAPSVK^1838^
11	^398^YSFCTDHAVLVQTR^411^	37	^1857^EINLSPDSTSVIVSGLM[147]VATK^1877^
12	^458^FGFCPMAAHEEICTTNEGVMYR^479^	38	^1878^YEVSVYALK^1886^
13	^504^GQWACIPYSQLR^515^	39	^1887^DTLTSRPAQGVVTTLENVSPPR^1908^
14	^670^GLTPGVIYEGQLISIQQYGHQEVTR^694^	40	^1887^DTLTSRPAQGVVTTLENVSPPRR^1909^
15	^785^YIVNVYQISEEGK^797^	41	^1926^TKTETITGFQVDAIPANGQTPVQR^1949^
16	^798^QSLILSTSQTTAPDAPPDPTVDQVDDTSIVVR^829^	42	^1928^TETITGFQVDAIPANGQTPVQR^1949^
17	^821^TQVSPTTLTAQWTAPSVK^838^	43	^1957^SYTITGLQPGTDYK^1970^
18	^830^WSRPQAPITGYR^841^	44	^1982^SSPVVIDASTAIDAPSNLR^2000^
19	^881^AVEENQESTPVFIQQETTGVPR^902^	45	^2001^FLTTTPNSLLVSWQAPR^2017^
20	^911^DLQFVEVTDVK^921^	46	^2008^SLLVSWQAPR^2017^
21	^938^VDVLPVNLPGEHGQR^952^	47	^2081^TDELPQLVTLPHPNLHGPEILDVPSTVQK^2109^
22	^1011^TVLVTWTPPR^1020^	48	^2161^LRPRPYLPNVDEEVQIGHVPR^2181^
23	^1054^NLQPGSEYTVTLMAVK^1069^	49	^2165^PYLPNVDEEVQIGHVPR^2181^
24	^1116^LGVRPSQGGEAPR^1128^	50	^2255^GVTYNIIVEALHNQR^2269^
25	^1254^ESAPISDTVIPEVPQLTDLSFVDITDSSIGLR^1285^	51	^2321^LTCQCLGFGSGHFR^2334^
26	^1365^FTNIGPDTMR^1374^	52	^2401^EYLGAICSCTCFGGQR^2416^
27	^1446^TGLDSPTGFDSSDVTANSFTVHWVAPR^1472^	53	^2452^TNTNVNCPIECFMPLDVQADRDDSRE^2477^

2D-LC-MS/MS of sciatic nerve samples was performed at day 7 after sham surgery or CCI. The sequence of the 53 identified FN peptides (with the total coverage over 35 %) is shown. The presence of the T*DELPQLVTLPHPNLHGPEI* ***LDV*** *PS*TVQK 2081–2109 peptide (#47) that included the CS1 sequence (italicized) implied that the 2477 residue long rat FN-CS1 splice variant (GeneBank #P04937.2) was present in our nerve samples

Using a specific antibody against the human FN-CS1 region (MAB1939, EMD Millipore), shown to cross-react with the murine antigen [26], we analyzed the FN-CS1 immunoreactivity in the sciatic nerve at day 7 post-CCI (Fig. 1d). Consistent with the previous reports [30], Schwann cells were the dominant source of FN-CS1. In addition, FN-CS1 was produced by vessel endothelial cells and CD68+ macrophages, which infiltrate sciatic nerve between 2 and 14 days post-injury [8]. Because Schwann cells deposit FN into the basement membrane [30] and Fig. 1d, single nerve fibers were teased out and immunostained for FN-CS1 at day 7 post-CCI for the subsequent detailed analyses (Fig. 1e). Consistent with the findings using pan-FN antibodies [30], the FN-CS1 immunoreactivity was dominant in the Schwann cell basement membrane of myelinated fibers, including the area immediately outside of the nodes of Ranvier.

### Acute CS1 therapy attenuates mechanical allodynia and IL-17A levels after CCI

**Fig. 2:**
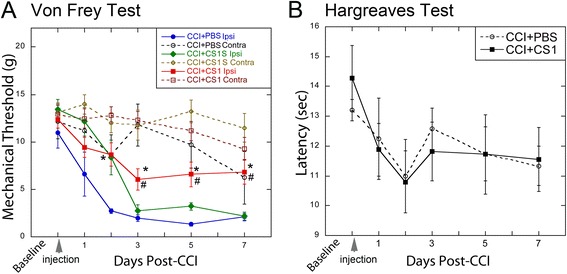
Acute CS1 therapy attenuates mechanical allodynia. The CS1 and scrambled (sCS1) peptides each dissolved in PBS (50 μg/ml in 5 μl) or PBS alone (5 μl) were administered by a single intraneural injection immediately after CCI (*arrow*). **a** von Frey testing for mechanical allodynia. Decline in the withdrawal threshold in the ipsilateral (*ipsi*) to CCI hind paw treated with PBS represents allodynia sustained for the study duration. sCS1 produced no significant change in the thresholds compared to PBS. In contrast, CS1-treated rats developed reduced sensitivity to tactile stimuli, compared to sCS1 (#) and PBS (*). Contralateral to injury, hind paws displayed no sensitivity to stimulus in either treatment group. The mean withdrawal thresholds (gram force; g) ± SEM of *n* = 6–17/group (* and #, *p* < 0.05, by ANOVA and Tukey’s post hoc test). **b** Hargreaves testing for thermal sensitivity. Withdrawal latency to thermal stimulation (radiant heat) decreased after CCI compared with prior to CCI (baseline). At the indicated days after CS1 and PBS injections, the sensitivity to thermal stimulation was not different between the groups. The mean paw withdrawal latency (seconds; s) ± SEM of *n* = 6/group

Competitive inhibition of the FN-CS1 binding to integrin α4 using a synthetic 25-residue-long CS1 peptide abrogated T cell trafficking into inflamed tissues [26, 28, 33, 34], required for the development of neuropathic pain phenotypes [44]. To analyze the effect of CS1 peptide therapy on pain-like behaviors, the wild-type CS1 peptide (DELPQLVTLPHPNLHGPEILDVPST) or the scrambled sCS1 peptide (EPDELQTGHVLSPLNHTPVLIPLDP) dissolved in PBS, or PBS alone was administered by a single intra-sciatic bolus injection in the CCI injury site, immediately after CCI surgery. Mechanical and heat sensitivity of the hind paw were then assessed once daily for one week (Fig. 2). A characteristic drop in the mechanical withdrawal threshold corresponding to robust mechanical allodynia occurred after CCI in animals receiving either sCS1 or PBS (Fig. 2a). In contrast, CS1 elevated the withdrawal threshold in response to mechanical stimulation compared with sCS1 or PBS. Neither CS1 nor sCS1 affected thermal hyperalgesia developed after CCI (Fig. 2b). Based on our results, we concluded that acute local CS1 peptide therapy delayed the development of mechanical, but not thermal, pain hypersensitivity by at least 1 week.

**Fig. 3:**
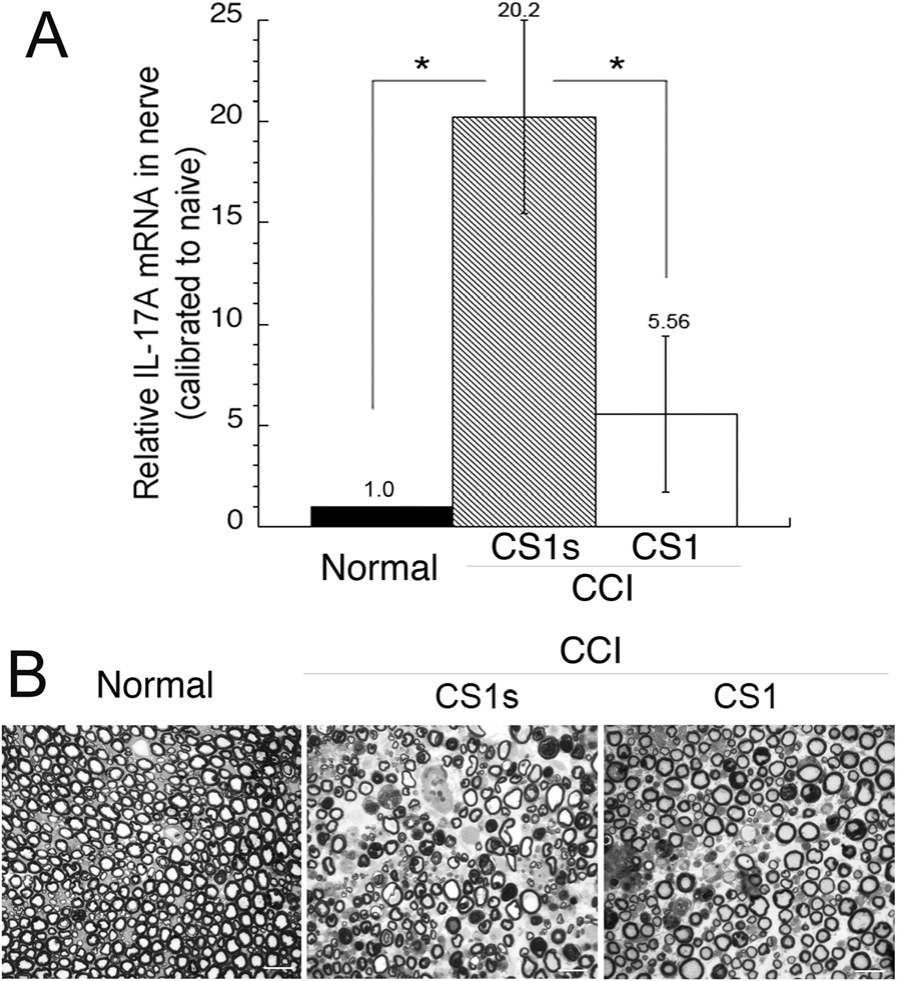
Acute CS1 therapy reduces IL-17A levels in CCI nerve. **a** *Taqman* qRT-PCR of IL-17A in sciatic nerve after acute intraneural CS1 or sCS1 treatment (50 μg/ml in 5 μl) performed at day 7 post-CCI after the completion of behavioral testing (Fig. 2). The mean relative mRNA ± SEM of *n* = 5/group normalized to GAPDH compared to naive nerve (*p < 0.05). **b** Methylene blue/azure II staining in 1-μm-thick sciatic nerve sections after acute intraneural CS1 or sCS1 treatment (50 μg/ml in 5 μl) performed at day 7 post-CCI, after the completion of behavioral testing (Fig. 2). Control nerve showed intact nerve morphology. CCI nerves displayed Wallerian degeneration (axonal degeneration, edema, myelin ovoids, and immune cell infiltration) after sCS1 treatment. In contrast, a greater number of uncompromised axons were observed in CCI nerves treated with CS1. Representative micrographs of *n* = 3/group. Scale bars, 20 μm

IL-17, a Th17 cell cytokine in CCI nerves [10, 13], selectively controls mechanical (but not thermal) pain hypersensitivity [13]. Given these comparable effects of the CS1 therapy and IL-17 gene deletion [13], we tested a model in which CS1 therapy acts by reducing IL-17A gene expression in the CCI nerves. Following the behavioral testing, we employed *Taqman* qRT-PCR to measure the IL-17A mRNA levels (Fig. 3a) and ultrastructural analysis (Fig. 3b) of the nerves exposed to CS1 or sCS1 peptides. The IL-17A mRNA was undetectable in naïve nerve (Fig. 3a), consistent with the report by others [10]. Accordingly, control nerve displayed the uniform morphology of intact axons (Fig. 3b). The IL-17A levels were significantly elevated in the injured nerves at day 7 post-CCI in the rats that received sCS1 (Fig. 3a). These nerves exhibited the characteristic features of Wallerian degeneration, including endoneurial edema, axon degeneration, the presence of myelin ovoids, and immune cell clusters (Fig. 3b). Both the IL-17A expression (Fig. 3a) and the features of degeneration (Fig. 3b) were significantly reduced in the animals that received CS1. These data suggested that acute CS1 therapy decreased a number of IL-17A+ (presumably, Th17) cells in the rodent neuropathy model.

### CS1 reduces ERK/MAPK activation in Schwann cells

**Fig. 4:**
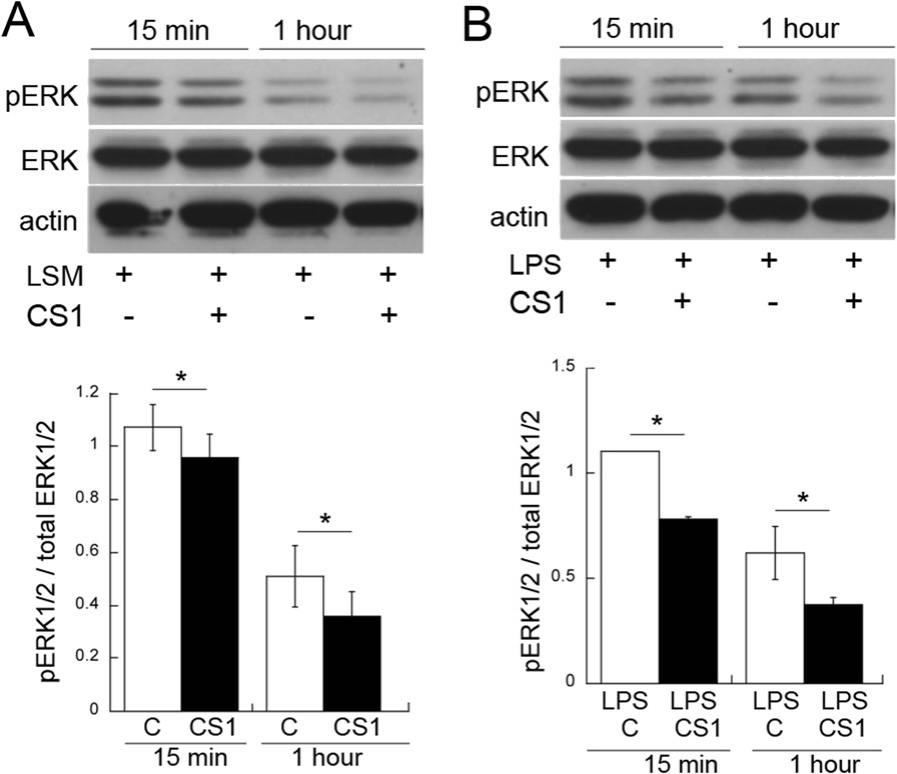
CS1 peptide inhibits Schwann cell activation. ERK1/2 immunoblotting in cultured Schwann cell lysates (11 μg total protein each) treated with CS1. Schwann cells were either starved in DMEM-1 % FBS for 24 h (**a**) or pre-treated with LPS (100 ng/ml) for 15 min (**b**) followed by CS1 (2.5 μg/ml) for 15 min or 1 h. The mean optical density of pERK1/2 to total ERK1/2 ratio based on four independent experiments (the graph, *p < 0.05)

Since Schwann cells are the dominant cell type expressing FN-CS1 post-CCI (Fig. 1), we evaluated the effect of CS1 peptide on Schwann cell activation (Fig. 4). Activation of the extracellular-signal-regulated kinase (ERK)/mitogen-activated protein kinase (MAPK) stress pathway contributes to neuropathic pain [8, 44] and can be stimulated in cultured primary Schwann cells by LPS treatment or low-serum medium (LSM) starvation [45]. CS1 inhibited the LPS- or starvation-stimulated activation of ERK (Fig. 4a). In addition, a short 15- to 60-min co-incubation of the CS1 peptide with the cells decreased pERK1/2 activation in Schwann cells that were stimulated with LPS for 15 min prior to CS1 (Fig. 4b). Based on these data, we argued that an intervention in the FN-CS1/α4β1 integrin interactions using the CS1 peptide reduced Schwann cell activation caused by stressful stimuli and or inflammation.

## Discussion

The mechanisms underlying chronic low-threshold pain phenotypes are poorly understood. Recent knowledge recognizes the involvement of adaptive immune response to physical nerve trauma, and specifically of Th cells, in chronic pain pathophysiology [4, 9–17]. It is interesting to note that adaptive immune cell modulators that regulate mechanical pain hypersensitivity do not elicit significant effect on heat pain hypersensitivity, including toll-like receptor-4 [19], myelin basic protein [17], IL-17 [13], IL-4 [18], and FN [20]. We have proposed that proteolytic release of the cryptic myelin auto-antigens initiates mechanical allodynia by selectively engaging mechanosensory myelinated A-afferents [17], involved in transducing the force of innocuous, low-threshold touch stimulation into nociceptive signal and the subsequent generation of mechanical allodynia after nerve injury [21, 22, 46–50]. The present study established that the alternatively spliced FN-CS1 isoform contributed to the selective mechanical allodynia onset in a rodent neuropathy model by regulating a content of IL-17A-expressing (presumably, Th17) cells at the nerve injury site.

Th17 cells (named after the cytokine IL-17 which they produce) are believed to be important in pain after nerve trauma [10, 13]. Although the IL-17A expression is generally restricted to a subtype of activated Th17 cells [51], including that in the CCI nerve [10, 13], the possibility of other endoneurial IL-17A+ cells in the post-CCI nerve cannot be excluded. The overall impediment of T cell function, achieved by the deletion of the CD4 gene [14] or recombinant activating gene-1 [10, 16] or by depletion in T cell production in athymic nude rats [9], improves resistance to both thermal and mechanical pain hypersensitivity. Thus, we suggest that the release of myelin auto-antigens initiates Th17 cell polarization and homing to myelinated afferents [17]. Subsequently, FN-CS1 mediates T cell adhesion, migration, and rolling along myelinated fibers [25–28, 52–54], which through secretion of algesic IL-17A [13] helps sustain the adaptive immune response and the persistent state of mechanical allodynia. At the later stage post-nerve damage, IL-17A release from Th17 cells may help sustain mechanical hypersensitivity by impeding Schwann-cell-mediated remyelination of sensory neurons [55].

In the injured peripheral nerve, the Schwann cell was the dominant source of FN-CS1, and interference with FN-CS1/α4β1 integrin interactions using the synthetic CS1 peptide repressed Schwann cell activation. Given that by interference with FN-CS1/α4β1 integrin interactions the CS1 peptide inhibited T cell homing, migration, and proliferation [25–28, 52–54], and that Schwann cell activation is central to immune cell recruitment into the injury site [8, 56], we suggest that upon Schwann cell activation, FN-CS1/α4β1 integrin binding (i) on endothelial cells facilitates T cell homing and transmigration across the blood-nerve barrier to the injured nerve and (ii) on Schwann cell basement membrane facilitates T cell adhesion, migration, and rolling along myelinated fibers. This latter mechanism offers the intriguing possibility of T cell rolling along the injured neuroaxis to the dorsal root ganglia and beyond. The present study does not distinguish myelinated efferent from afferent fibers within the mixed (motor/sensory) sciatic nerve. However, nociceptive factors released from the damaged efferents contribute to mechanical pain hypersensitivity [57]. We did not rule out the FN-CS1 deposition on Schwann cell basement membrane of unmyelinated afferent C-fibers. Within each Schwann cell basement membrane lie a bundle of 2–10 unmyelinated C-fibers (Remak bundle), yet only one A-fiber [30], suggesting more limited T cell access to individual heat-sensitive C-fibers compared with mechanosensitive A-fibers. Notably, FN-CS1 also deposited on the Schwann cell basement membrane immediately outside of the nodes of Ranvier, the site of the likely T cell contact post-CCI [17].

The sustained effect of the acute and local CS1 therapy to attenuate mechanical but not thermal pain hypersensitivity has also been observed after the acute and local (intra-spinal cord) CS1 injection after spinal cord dorsal column hemisection [20]. This analgesic CS1 effect sustained over a long (25-week) observation period and related to the reduced serotonin 5-HT system and monocyte content in the damaged spinal cord [20], although the changes in adaptive immune response remain to be assessed in that model. In addition to these localized effects to activate Schwann cells and recruit immune cells into the injury site, FN promoted mechanical allodynia by activating ATP-gated ion channel P2X4 purino-receptor on spinal microglia after spinal nerve injury [58]. However, when considering targeting the FN-CS1/α4β1 integrin binding by using CS1 peptide or other approaches (e.g., α4 integrin-neutralizing antibody, natalizumab) for neuropathy management [59], adverse effects on immune cell function or Schwann-cell-mediated outgrowth of sensory neurons expressing α4β1 integrin [60, 61] should be assessed with caution.

In the injured peripheral nerve, FN undergoes structural modifications by the complete or partial inclusion or exclusion of the alternatively spliced type IIICS/V domain [31, 32]. V120, V95, and V0 splice forms of FN [42] and (Fig. 1a) exist in both naïve and injured nerves [31, 32, 43], with about a 20–50 % increase in the CS1-containing V120 following injury [60]. The levels of high molecular weight FN species escalated day 7 post-CCI, the time of active T cell recruitment [4, 9, 10, 12]. Proteolysis of FN by matrix metalloproteinases (MMP)-9 and MMP-14 (Additional file 1: Figure S1), whose activity is significantly enhanced in nerve injury [17, 56, 62], may contribute to the additional molecular diversity of FN. The Leu-Asp-Val (LDV) sequence that is present in the nerve FN-CS1 binds α4β1 integrin on T cells with 20-fold more efficiency relative to the Arg-Gly-Asp (RGD) sequence [42]. In addition to challenging the FN-CS1/α4β1 integrin binding, the CS1 peptide may act by interfering with α4β1 integrin interactions with other ligands, such as vascular cell adhesion protein 1 [27, 63].

## Conclusion

Expression of the FN-CS1 splice variant in peripheral nerve was observed in a painful rodent neuropathy model. FN-CS1, an extracellular glycoprotein, deposited into the blood-nerve barrier and the Schwann cell basement membrane underlying myelinated mechanosensory A-afferents. Interference with the FN-CS1/α4β1 integrin interaction using CS1 peptide therapy reduced the levels of the algesic IL-17A and rescued the animals from the nerve-injury-induced mechanical pain hypersensitivity.

## Additional file

Additional file 1: Figure S1. Prediction and ranking of the MMP cleavage sites. The CS-1 region of the fibronectin peptide sequence. The CS-1 sequence is present within the FN-CS1 splice variant. The arrows point to the P1 residues of the putative cleavage sites. The data suggest the presence of the multiple MMP cleavage sites in the CS-1 sequence including the cleavage site for MT1-MMP/MMP-14 (LPHP-NLH and GPEI-LDV).

## Abbreviations

CCI: chronic constriction injury
CS1: connecting segment 1 peptide
DAPI: 4′,6-diamidino-2-phenylindole
ERK: extracellular-signal-regulated kinase
FN: fibronectin
IL: interleukin
LPS: lipopolysaccharide
MAPK: mitogen-activated protein kinase
sCS1: scrambled CS1 peptide
Th cell: T helper cell
