# Supplementary material for: The alternatively spliced fibronectin CS1 isoform regulates IL-17A levels and mechanical allodynia after peripheral nerve injury
Source: J Neuroinflammation. 2015 Sep 4;12:158. doi: 10.1186/s12974-015-0377-6 (PMC4559385; doi:10.1186/s12974-015-0377-6)
Supplement: Additional file 1: Figure S1. — Prediction and ranking of the MMP cleavage sites. The CS-1 region of the fibronectin peptide sequence. The CS-1 sequence is present within the FN-CS1 splice variant. The arrows point to the P1 residues of the putative cleavage sites. The data suggest the presence of the multiple MMP cleavage sites in the CS-1 sequence including the cleavage site for MT1-MMP/MMP-14 (LPHP-NLH and GPEI-LDV). [file 12974_2015_377_MOESM1_ESM.docx]

| 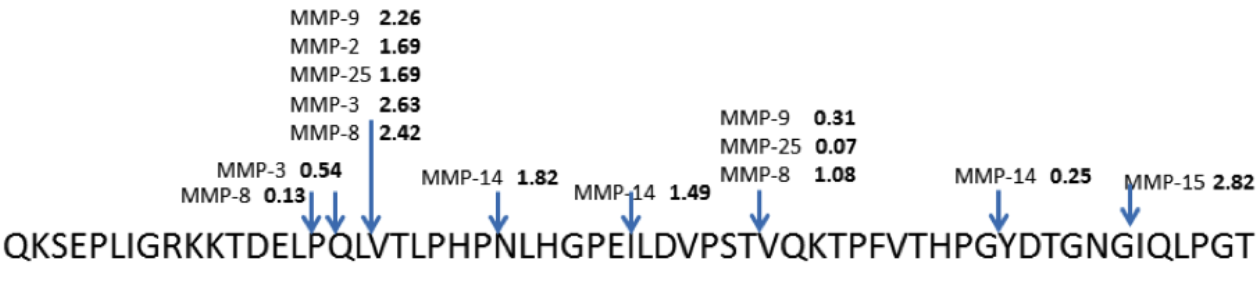 |
| --- |
| **Suppl Fig. S1.** **Prediction and ranking of the MMP cleavage sites.** The CS-1 region of the fibronectin peptide sequence. The CS-1 sequence is present within the FN-CS1 splice variant. The arrows point to the P1 residues of the putative cleavage sites. The data suggest the presence of the multiple MMP cleavage sites in the CS-1 sequence including the cleavage site for MT1-MMP/MMP-14 (LPHP-NLH and GPEI-LDV). |

**Prediction and ranking of the MMP cleavage sites.** To predict the cleavage sites for the individual MMPs in the fibronectin sequence, we used the positional weight matrices (PWM) computer program we developed [(Ratnikov et al, PNAS USA 2014, 111(40): E4148-55) and http://[www.CleavPredict.SanfordBurnham.org](http://www.CleavPredict.SanfordBurnham.org)]. The program determines the contribution of each amino acid residue at each of the P3-P2’ positions to the efficiency of the protein proteolysis by a proteinase and assigns a numerical score to every peptide bond in the protein sequence. A high PMW score indicates high probability of the proteolysis. Conversely, a low score suggests low probability of the cleavage of the particular scissile bond by the proteinase.
